# Genome Sequence, Assembly, and Characterization of the Antagonistic Yeast *Candida oleophila* Used as a Biocontrol Agent Against Post-harvest Diseases

**DOI:** 10.3389/fmicb.2020.00295

**Published:** 2020-02-25

**Authors:** Yuan Sui, Michael Wisniewski, Samir Droby, Edoardo Piombo, Xuehong Wu, Junyang Yue

**Affiliations:** ^1^Chongqing Key Laboratory of Economic Plant Biotechnology, Collaborative Innovation Center of Special Plant Industry in Chongqing, College of Forestry and Life Science, Institute of Special Plants, Chongqing University of Arts and Sciences, Yongchuan, China; ^2^U.S. Department of Agriculture-Agricultural Research Service, Kearneysville, WV, United States; ^3^Department of Postharvest Science, Agricultural Research Organization, Volcani Center, Bet Dagan, Israel; ^4^Department of Agricultural, Forestry and Food Sciences, University of Turin, Turin, Italy; ^5^Department of Plant Pathology, China Agricultural University, Beijing, China; ^6^School of Food and Biological Engineering, Hefei University of Technology, Hefei, China

**Keywords:** biocontrol agent, *Candida oleophila*, genome assembly, genome annotation, post-harvest disease management

## Abstract

*Candida oleophila* is an effective biocontrol agent used to control post-harvest diseases of fruits and vegetables. *C. oleophila* I-182 was the active agent used in the first-generation yeast-based commercial product, Aspire^®^, for post-harvest disease management. Several action modes, like competition for nutrients and space, induction of pathogenesis-related genes in host tissues, and production of extracellular lytic enzymes, have been demonstrated for the biological control activity exhibited by *C. oleophila* through which it inhibits post-harvest pathogens. In the present study, the whole genome of *C. oleophila* I-182 was sequenced using PacBio and Illumina shotgun sequencing technologies, yielding an estimated genome size of 14.73 Mb. The genome size is similar in length to that of the model yeast strain *Saccharomyces cerevisiae* S288c. Based on the assembled genome, protein-coding sequences were identified and annotated. The predicted genes were further assigned with gene ontology terms and clustered in special functional groups. A comparative analysis of *C. oleophila* proteome with the proteomes of 11 representative yeasts revealed 2 unique and 124 expanded families of proteins in *C. oleophila*. Availability of the genome sequence will facilitate a better understanding the properties of biocontrol yeasts at the molecular level.

## Introduction

The use of biocontrol yeasts to manage post-harvest diseases of fruits and vegetables has been actively investigated (Droby et al., 2016; Wisniewski et al., 2016; Contarino et al., 2019). Among the antagonistic yeasts, *Candida oleophila* has been reported to be an effective biocontrol agent against several post-harvest pathogens that cause decay in a variety of fruits, including apple (El-Neshawy and Wilson, 1997), grapefruit (Droby et al., 2002), kiwifruit (Wang et al., 2018), banana (Bastiaanse et al., 2010), and pear (Nie et al., 2019). *C. oleophila* I-182 was the active agent in the first yeast-based commercialproduct, Aspire^®^, for the management of post-harvest diseases (Droby et al., 1998). Although the product is no longer available, another strain, *C. oleophila* strain O, has since been used to develop a new post-harvest biocontrol product, Nexy^®^ (Massart and Jijakli, 2014). Several modes of action for the biocontrol activity of *C. oleophila* I-182 have been demonstrated, including competition for nutrients and space (El-Neshawy and Wilson, 1997), induction of pathogenesis-related genes and proteins (Droby et al., 2002; Liu et al., 2013), oxidative stress tolerance (Wang et al., 2018), production of extracellular lytic enzymes (Bar-Shimon et al., 2004) and superoxide anion production (Macarisin et al., 2010). Additionally, a suppressive-subtractive hybridization (SSH) cDNA library that identified several antioxidant genes associated with biocontrol activity and stress tolerance in *C. oleophila* I-182 was also constructed (Liu et al., 2012). Information on its genome sequence, assembly, and annotation, however, is currently lacking.

The genome sequences of two biocontrol yeasts *Metschnikowia fructicola* (strains 277 and AP47) (Piombo et al., 2018), and a plant growth-promoting endophytic yeast, *Rhodotorula graminis* (strain WP1) (Firrincieli et al., 2015) have been previously reported. Genome sequence information is a valuable reference for determining the sequences of putative “biocontrol/growth-promoting related” genes in different species of yeasts, characterizing gene clusters with known and unknown functions, as well as for identifying global changes in the expression of gene networks rather than just specific, targeted genes. A full genome sequence also enables one to conduct comparative genomic analyses among closely related yeast species that do not exhibit biocontrol properties (Massart et al., 2015).

In the present study, the whole genome of *C. oleophila* strain I-182 was sequenced and assembled using a combination of both PacBio and Illumina sequencing platforms. Results indicate that the size of the *C. oleophila* genome is approximately 14.13 Mb and contains 5,615 protein-encoding genes. The genome sequence, assembly, and annotation can be used to further elucidate the molecular mechanism underlying the biocontrol activity of yeast antagonists against several higher fungi responsible for causing decay in harvested fruits and vegetables.

## Materials and Methods

### Sample Collection and Cell Culture

The type-culture of the biocontrol yeast, *C. oleophila* I-182 (ATCC^®^ MYA-1208^TM^), originally isolated from the surface of tomato fruit (Wilson et al., 1993), was grown in a yeast-peptone-dextrose (YPD) broth (10 g of yeast extract, 20 g of peptone, and 20 g of dextrose in 1 L of distilled water). Twenty milliliters of YPD broth was placed in 50-mL conical flasks and inoculated with *C. oleophila* at an initial concentration of 10^5^ cells/mL. Yeast cultures were incubated at 25°C for 48 h at 200 r.p.m. The yeast cells were pelleted by centrifugation at 8,000 *g* for 2 min, and subsequently washed three times with sterile distilled water to remove any residual medium. Approximately, 2 g (fresh weight) of yeast cells were used for DNA extraction as described below.

### DNA Extraction and Genome Sequencing

PacBio sequencing-genomic DNA of *C. oleophila* was prepared as previously described (Pirone-Davies et al., 2015). High molecular weight (HMW) genomic DNA was extracted and sheared into fragments approximately 20 kb in size using g-Tubes (Covaris, Inc., Woburn, MA, United States) according to the manufacturer’s instructions. The fragment ends were subsequently repaired and ligated with the connector of a hairpin structure to form a dumbbell structure called SMRTbell. The SMRTbell library was constructed using a DNA Template Prep Kit 1.0 and the 20-kb insert library protocol (Pacific Biosciences, Menlo Park, CA, United States). Size selection was performed with BluePippin (Sage Science, Beverly, MA, United States). The resulting library was sequenced using P6/C4 chemistry on a PacBio^®^ RS II Sequencer System (Pacific Biosciences), with a 240-min collection protocol along with stage start.

For next-generation sequencing (NGS), genomic DNA was extracted and fragmented into random sizes using Covaris^TM^ S2 (Covaris, Inc.). The overhangs generated from fragmentation were converted into blunt ends using Illumina’s Genomic DNA Sample Preparation kit (Illumina, San Diego, CA, United States). After adding an ‘A’ base to the 3′ end of the blunt phosphorylated DNA fragments, adapters were ligated to the ends of the DNA fragments. The desired DNA fragments were selected by gel-electrophoresis and amplified by PCR. Two, paired-end Illumina libraries with insert sizes of 300 and 10,000 bp were prepared and subsequently sequenced on an Illumina HiSeq 2500 system (Illumina).

### Genome Assembly and Error Correction

Prior to genome assembly, the size of the genome, degree of heterozygosity and the level of gene duplication were estimated by *k*-mer analysis using GenomeScope (Vurture et al., 2017). The genome was assembled using a *de novo* approach. Illumina reads of different insert size were first trimmed with Trimmomatic v. 0.36 to remove low quality reads (Bolger et al., 2014). Sequence data obtained from the PacBio long-read sequencing were analyzed using the SMRT Link pipeline version 5.1.0 and the HGAP program version 3.0 (Chin et al., 2013). In the HGAP protocol, the parameters of minimum sub-read length cutoff and target coverage were set at 5,000 kb and 20X, respectively. The obtained contigs were corrected and assembled using Canu version 1.7 (Koren et al., 2017). Finally, the assembly was polished using the Quiver tool (Chin et al., 2013) and further corrected using the high-quality, cleaned Illumina reads and Pilon version 1.22 (Walker et al., 2014).

### Genome Annotation

After obtaining the assembled genome, the distribution of functional elements was primarily annotated using homology-based predictions. The repeat-masked genome sequences were identified by RepeatMasker (Saha et al., 2008), and protein-coding genes were predicted by GeneScan (Burge and Karlin, 1997). A homologous sequence search was performed through alignment with the yeast S288c genome downloaded from *Saccharomyces* genome database (SGD^1^) using the BLASTN program with an *E*-value cutoff 1*e*-5. Annotation of the predicted genes was performed by querying against a number of nucleotide and protein databases, including non-redundant (nr), Swiss-Prot, TrEMBL, KEGG, COG, P450, VFDB, ARDB, TF, CAZY, PHI, IPR, and T3SS (*E*-value = 1*e*-5). Gene ontology (GO) terms were assigned to the annotated genes using the Blast2GO pipeline (Ashburner et al., 2000). Conserved domains within the predicted protein sequences of *C. oleophila* were identified by comparison against datasets from the Pfamand InterPro databases. Secondary metabolite clusters were predicted using the antiSMASH tool (Weber et al., 2015). Non-coding RNAs were also identified using the Infernal tool (Nawrocki and Eddy, 2013). To ensure the biological relevance, the results with the highest quality alignment were selected and retained for the annotation of all of the identified genes.

### Gene Family Identification and Genome Evolution

The OrthoFinder package ver. 2.2.7 (Emms and Kelly, 2015) was used to identify and compare gene families present in *C. oleophila* I-182 and 11 other representative yeast species, including *Candida maltosa* Xu316, *Candida tenuis* ATCC 10573, *Debaryomyces hansenii* CBS 767, *Lachancea thermotolerans* CBS 6340, *M. fructicola* CBS 8853, *Pichia kudriavzevii* str. 129, *Pichia membranifaciens* NRRL Y-2026, *Saccharomyces cerevisiae* S288c R64-1-1, *Tetrapisispora phaffii* CBS 4417, *Torulaspora delbrueckii* CBS 1146, and *Wickerhamomyces anomalus* NRRL Y-366-8. The protein sequences of these species were downloaded from the EnsemblFungi database^2^. Species-specific proteins, as well as their protein families, were determined based on their presence or absence in a given species. The dynamic evolution (expansion and contraction) of orthologous protein families was explored with Computational Analysis of gene Family Evolution (Café 3.1) (de Bie et al., 2006) using probabilistic graphical models. Evolutionary relationships among the 12 examined yeast species were resolved with the Randomized Accelerated Maximum Likelihood package (RAxMLversion 8) (Stamatakis, 2006) using 538 single-copy and high-quality orthologous members. The generated phylogenetic tree was visualized using MEGA version 10 (Kumar et al., 2018).

## Results and Discussion

### Sequence Data

The availability of the whole genome sequence of microbial biocontrol agents will facilitate a more comprehensive understanding of the mode of action at a molecular level (Druzhinina et al., 2011). In the present study, an assembly of the genome of *C. oleophila* I-182 was achieved by combining the long but relatively low-quality PacBio reads, with the shorter but higher quality Illumina reads using a complex approach. As a result, a high-quality genome sequence of *C. oleophila* I-182 was constructed. The assembled gapless and near-complete genome is equivalent in length to that of the model yeast species, *S. cerevisiae* S288c (∼12.2 Mb^3^), but much less than the size of another biocontrol species *M. fructicola* (∼26 Mb; Piombo et al., 2018). Three SMRT cells were constructed and sequenced on the PacBio RS II Sequencer providing up to 1,516 Mb of sequence data. A total of 103,064 reads with a mean and median length of 14,713 and 21,808 bp, respectively were generated. Illumina sequencing technology of two paired-end Illumina libraries with insert sizes of 300 and 10,000 bp was also utilized producing a total of862 and 1,259 Mb of raw sequence data for the small and large fragments, respectively comprising 5,749,278 and 8,397,144 reads respectively. After removal of the adaptor sequences and filtering out low quality reads, approximately 741 and 699 Mb high-quality cleaned sequences were obtained for the small and large fragments (Table 1). The raw sequencing data have been deposited at the Sequence Read Archive of NCBI database, under the accession number PRJNA511409^4^.

**TABLE 1:** Summary of the sequencing data obtained with PacBio and Illumina technology and used for the genome assembly of *C. oleophila* I-182.

**Sequencing**	**PacBio RS II**	**Illumina**	
**platform**			
		**300 bp library**	**10,000 bp library**
Raw data	1,516 Mb	862 Mb	1,259 Mb
Clean data	1,509 Mb	741 Mb	699 Mb
Read number	103,064	5,749,278	8,397,144
			

### Genome Size and Assembly

A *k*-mer analysis of the sequence data indicated that the estimated size of the *C. oleophila* genome was 14.73 Mb. Thus, the clean data generated from the PacBio and Illumina sequencing platforms represented 107 × and 101 × coverage of the genome, respectively.

The clean, high-quality sequences from each platform were first independently assembled and optimized after multiple adjustments. The two assemblies were then merged to improve contiguity using the Quickmerge tool (Chakraborty et al., 2016). This resulted in the construction of a high-quality genome consisting of 10 contigs with an N50 of 1,848,245 bp. The resulting contigs were then further assembled into 8 scaffolds by mapping the genome against the yeast S288c reference genome (SGD^5^). Thefinal size of the *C. oleophila* genome in the released version was 14.13 Mb. Details of the genome assembly statistics are presented in Table 2.

**TABLE 2:** The details of genome assembly statistics for *C. oleophila*.

**Assembly**	**Scaffold**	**Contig**
Total number	8	10
Total length	14,129,745	14,129,104
N50 length	2,030,489	1,848,245
N90 length	1,455,442	1,455,442
Maximum length	3,488,600	2,315,880
Minimum length	74,302	1,795
GC content	39.39	39.39

### Gene Prediction and Annotation

Functional genes were predicted based on homologous sequence searching. As a result, 5,615 protein-encoding genes with 8,004 exons were identified. The average length of these gene sequences is 1,683 bp, and the average number of exons per gene is 1.43. Of the 5,615 genes identified in the *C. oleophila* genome, 4,779, 2,839, 3,162, 3,745, and 727 were aligned to the nr, Swiss-Prot, KEGG, GO, and COG databases, respectively, using an *E*-value cutoff of 1*e*-5. The statistics regarding gene annotation from the P450, VFDB, ARDB, TF, TrEMBL, CAZY, PHI, IPR, and T3SS databases are also listed in Table 3. After eliminating the redundancy of genes listed in different databases, a total of 5,356 genes were annotated at least once, covering up to 95.39% of the identified gene sequences.

**TABLE 3:** Annotation of the predicted genes using a variety of databases.

**Database**	**Full name**	**Count**	**%**
nr	Non-redundant protein database	4,779	85.11
Swiss-Prot	The UniProtKB/Swiss-Prot database	2,839	50.56
KEGG	Kyoto encyclopedia of genes and genomes	3,162	56.31
GO	Gene ontology	3,745	66.69
COG	Cluster of orthologous groups of proteins	727	12.94
P450	Fungal cytochrome P450	349	6.21
VFDB	Virulence factors of pathogenic bacteria	37	0.65
ARDB	Antibiotic resistance genes database	1	0.01
TF	Transcription factor database	255	4.54
TrEMBL	Translated EMBL nucleotide sequence data library	4,751	84.61
CAZY	Carbohydrate-active enzymes database	103	1.83
PHI	Pathogen host interactions	468	8.33
IPR	The interpro database	4,881	86.92
T3SS	Type III secretion system effector protein	2,072	36.9
Total		5,356	95.38

**FIGURE 1:**
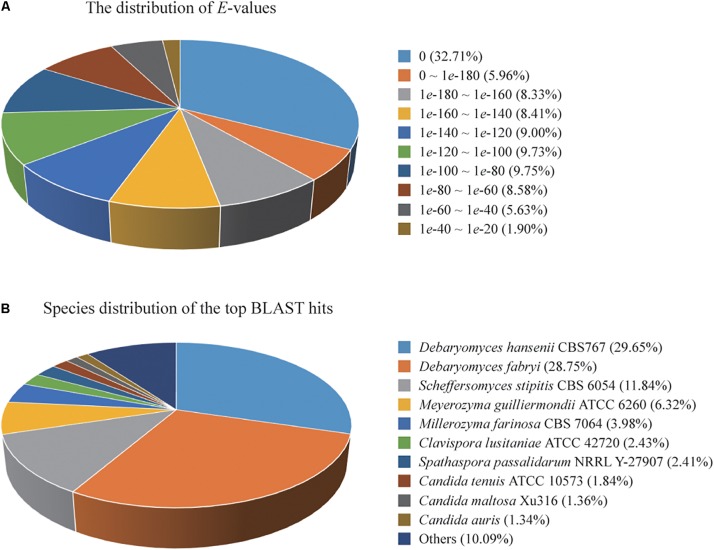
**(A)** Percent distribution of *E*-value from the alignment of *Candida oleophila* predicted genes with available sequences in the nr database. **(B)** Species distribution of the top BLAST hits for the best alignment of *C. oleophila* predicted genes against the nr database.

A total of 4,779 of the annotated genes were present in the nr database, accounting for approximately 89.23% of the total number of annotated genes. A statistical analysis of the distributed *E*-value revealed that 83.89% of the mapped sequences have strong homologies (*E*-value < 1*e*-80) to sequences available in the nr database (Figure 1A). The species distribution of the top BLAST hits for the best alignment in the nr database is presented in Figure 1B. The species with the highest percentage of homologous genes were *D. hansenii* CBS767 (29.65%), *Debaryomyces fabryi* (28.75%), *Scheffersomyces stipitis* CBS 6054 (11.84%), *Meyerozyma guilliermondii* ATCC 6260 (6.32%), *Millerozyma farinosa* CBS 7064 (3.98%), *Clavispora lusitaniae* ATCC 42720 (2.43%), *Spathaspora passalidarum* NRRL Y-27907 (2.41%), *C. tenuis* ATCC 10573 (1.84%), *C. maltosa* Xu316 (1.36%), and *Candida auris* (1.34%).

**FIGURE 2:**
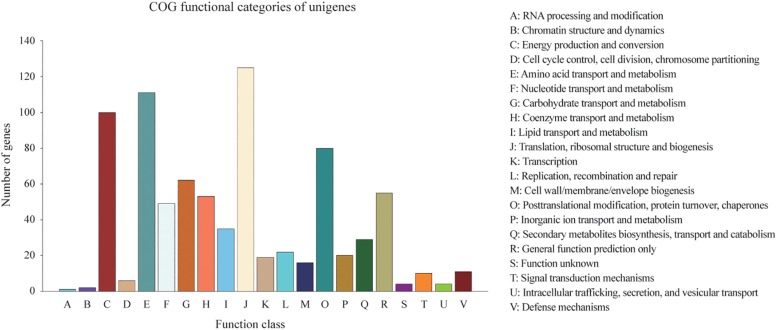
Distribution of 727 predicted genes in *C. oleophila* and 21 different COG functional categories.

Homologies within the Swiss-Prot database were also assessed by manual curation, consequently representing high quality and accuracy. As a result, 2,839 genes were identified and annotated within the Swiss-Prot database, all of which had also been identified and annotated within the nr database. Additionally, 3,162 and 727 genes were mapped to 372 KEGG pathways and 21 COG categories, respectively. The KEGG pathways for ‘metabolic pathways’ represented the largest group, followed by ‘biosynthesis of secondary metabolites,’ ‘biosynthesis of antibiotics,’ ‘microbial metabolism in diverse environments,’ and ‘biosynthesis of amino acids’ (Supplementary Table S1). The categories of genes most frequently mapped to the21 COG categories, included ‘translation, ribosomal structure, and biogenesis,’ ‘amino acid transport and metabolism,’ ‘energy production and conversion,’ ‘post-translational modification, protein turnover, chaperones,’ and ‘carbohydrate transport and metabolism’ (Figure 2).

**FIGURE 3:**
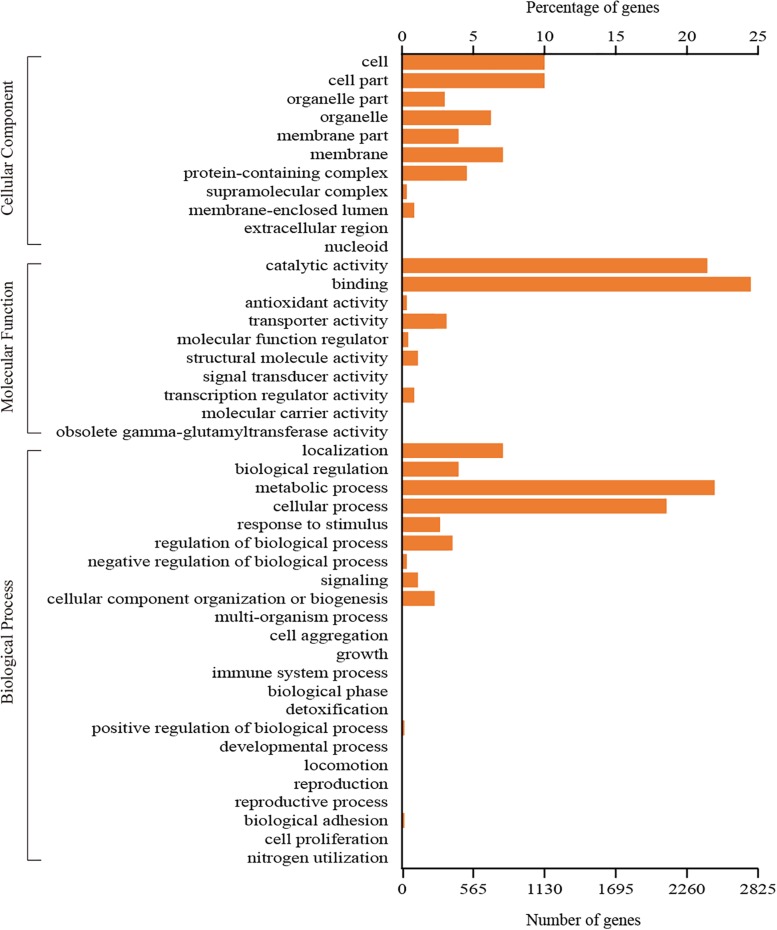
GO classification of all the identified genes in *C. oleophila* was summarized as three main categories: biological process, molecular function and cellular component.

A total of 3,745 genes could be assigned to at least one GO category using the Blast2GO pipeline. Among them, 2,618 genes were classified in the biological process category, 1,400 genes were classified in the cellular component category, and 3,152 genes were classified in the molecular function category. A total of 44 functional GO terms were annotated (Figure 3). For each of the three main categories, the dominant GO terms were ‘metabolic process’ (in ‘biological process’), ‘cell or cell part’ (in ‘cellular component’) and ‘binding’ (in ‘molecular function’). In contrast, relatively few genes representing ‘locomotion’ (in ‘biological process’), ‘nucleoid’ (in ‘cellular component’) and ‘molecular carrier activity’ (in ‘molecular function’) were identified.

In addition to protein-encoding genes, non-coding sequences are also involved in many cellular processes. In the present study, rRNA, tRNA, sRNA, snRNA, and miRNA sequences present in *C. oleophila* were identified using the Infernal tool (Nawrocki and Eddy, 2013). The statistics of their copy number and sequence length is shown in Table 4. Additionally, a total of 431.35 kb repeat sequences were also identified in the genome of *C. oleophila* by RepeatMasker (Saha et al., 2008).

**TABLE 4:** Statistics of different types of ncRNA in the *C. oleophila* genome.

**Type**	**Copy**	**Average length (bp)**	**Total length (bp)**	**% in Genome**
tRNA	246	79	19,159	0.1356
rRNA	19	1,900	36,107	0.2555
sRNA	100	72	7,219	0.0511
snRNA	38	110	4,162	0.0295
miRNA	132	56	7,417	0.0525

The high integrity of the assembled genome enabled the identification and annotation of a large number of protein-coding genes through the use of multiple annotation approaches. A comparison of annotated genes between I-182 and S288c revealed a number of variations in protein-coding genes, which could be relevant to functional properties and gene evolution in *C. oleophila*.

### Gene Families and Evolution

**FIGURE 4:**
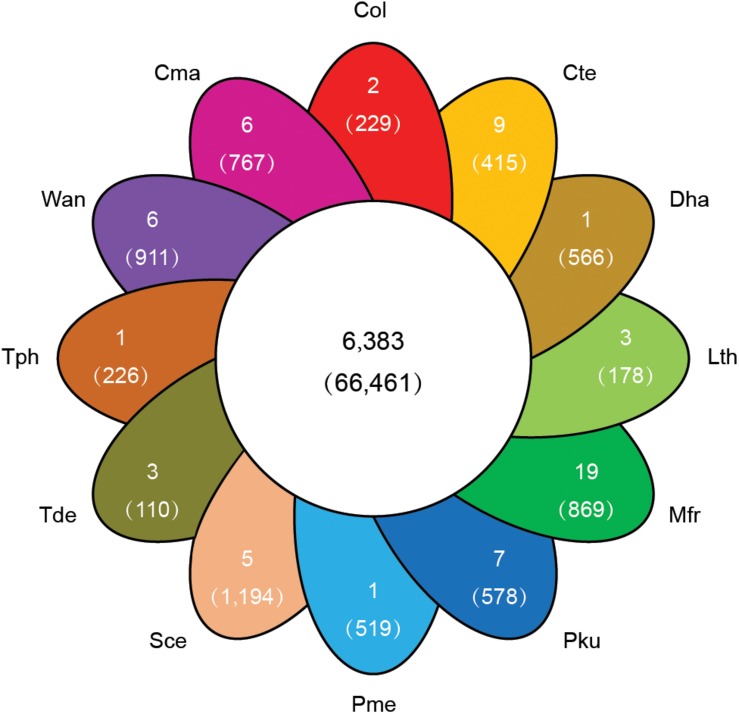
Venn diagram indicating the number of shared and specific gene families among *C. oleophila* and 11 other representative yeast species. The number in the middle white circle indicates the number of shared families (no parentheses) and the number of shared genes (parentheses). In each of the colored section the number of unique gene families (no parentheses) is indicated and the number of genes within the species-specific families (parentheses) is indicated. Three-letter acronym for the abbreviation of each species.

To explore the genomic basis of species adaptation during evolution, the identified proteome of *C. oleophila* was compared to the proteome of 11 other representative yeasts. The yeast species were selected based on their use as a model organism (*S. cerevisiae*) or because of their reported use as a biocontrol agent against a variety of plant diseases. The latter includes *C. maltosa*, *C. tenuis*, *D. hansenii*, *L. thermotolerans*, *M. fructicola*, *P. kudriavzevii*, *P. membranifaciens*, *T. delbrueckii*, *T. phaffii*, and *W. anomalus*. The analysis identified a total of 6,383 orthologous protein families comprising 66,461 proteins. The comparison further identified 36,833 proteins belonging to 2,529 families that were shared among all 12 yeasts, representing a core set of ancestral clusters. In contrast, 229 proteins belonging to two different families were found to be specific to *C. oleophila*, suggesting that they may play a unique biological function or have a specific phytochemical property within this species (Figure 4). Functional enrichment analysis based on the GO annotation revealed that the specific proteins in *C. oleophila* tended to possess NADH dehydrogenase (ubiquinone) activity (GO:0008137) and glutathione peroxidase activity (GO:0004602) (Supplementary Table S2).

**FIGURE 5:**
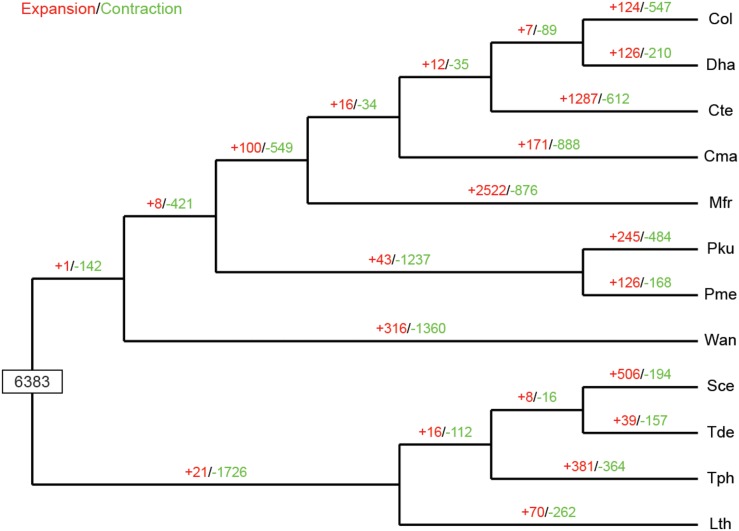
Expansion and contraction of gene families among the 12 yeast species. Phylogenetic tree was constructed based on 538 high-quality 1:1 single-copy orthologous genes. The numerical values on each branch of the tree represent gene families undergoing gain (red) or loss (green) events. Gene families predicted in the most recent common ancestor (MRCA) was 6,383. Three-letter acronym for the abbreviation of each species name.

The expansion and contraction of gene families in yeast species are crucial driving forces of lineage splitting and physiological diversification (Papp et al., 2003). Therefore, gene families that had experienced discernible changes and adaptive evolution along divergent branches were characterized. Particular emphasis was placed on *C. oleophila* as representing a biocontrol agent. A phylogenetic analysis was also performed to discern the evolutionary relationships among multiple species. Results indicated that among the 6,383 gene families inferred to be present in the most recent common ancestor (MRCA) of the 12 examined species of yeasts, 124 families were expanded in *C. oleophila* (Figure 5). GO annotation of 346 genes from 69 families with significant expansions (*P* < 0.05) revealed that they were primarily enriched in functional categories related to cell adhesion (in ‘biological process’) and coenzyme binding (in ‘molecular function’), which provided interesting information on the metabolic network architecture in this species (Supplementary Table S3).

Functional analysis of the specific and expanded gene families could potentially provide important information on the biocontrol mechanisms of *C. oleophila*. For example, yeast biofilms formed by the secretion of a extracellular matrix that provides protection and helps yeast adhere to the surface of host cells and tissues will directly influence environmental persistence and attachment capability, and ultimately biocontrol activity (Freimoser et al., 2019). In addition, enzymes involved in the antioxidant system of yeast, such as glutathione peroxidase, catalase, and superoxide dismutase, have been reported to be associated with biocontrol efficacy in *C. oleophila* (Liu et al., 2012), as well as several other yeast, including *Cystofilobasidium infirmominiatum* (Liu et al., 2011), and *Pichia caribbica* (Li et al., 2014).

### Enzymes Involved in Carbohydrate Metabolism

The cell walls of vascular plant hosts consist of a complex network of carbohydrate components, including cellulose, hemicellulose, and pectin. These carbohydrates have the potential to be catalyzed into oligomers and simple monomers that can be used as nutrients by microbes (Cantarel et al., 2009). Bacteria and fungi have evolved a variety of carbohydrate-active enzymes (CAZymes) in response to their interaction with their plant hosts (Kolton et al., 2013). Our analysis indicates that *C. oleophila* encodes 103 genes representing CAZymes. These include54 polysaccharide lyases (PLs), 37 glycosyl transferase (GTs), 1 glycoside hydrolases (GHs), 5 carbohydrate esterases (CEs), and 5 carbohydrate-binding modules (CBMs). All of the identified CAZymes have the potential to be involved in the degradation of the cell walls, which is an important attribute of yeasts as biocontrol agents against fungal pathogens. For instance, *CoEXG1*, which encodes a secreted 1,3-β-glucanase in *C. oleophila* I-182, was cloned, and its role in biocontrol was characterized (Segal et al., 2002; Yehuda et al., 2003; Bar-Shimon et al., 2004). Other antagonistic fungi, such as *Aureobasidium pullulans* JYC1291, *Galactomyces candidum* JYC1146, and *Trichoderma harzianum* CECT 2413, produce and secrete different types of CAZymes, that play an important functional role in the degradation of the cell wall of fungal pathogens (Ait-Lahsen et al., 2001; Chen et al., 2018). Whether the CAZymes produced by biocontrol agents have a detrimental effect on host tissues, however, has not been explored. Notably, there are no existing reports of selected biocontrol yeast species causing infection in the hosts they protect or related hosts, although admittedly, comprehensive studies have not been conducted.

### Secondary Metabolite Clusters

**FIGURE 6:**
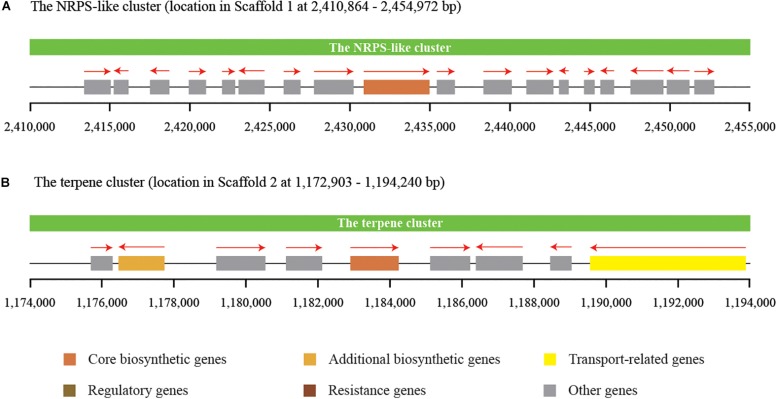
Identification of two distinct secondary metabolite clusters in the genome of *C. oleophila.* **(A)** The non-ribosomal peptide synthetase (NRPS)-like cluster is composed of 18 functional genes. **(B)** The terpene cluster is composed of nine functional genes. The rectangle denotes a functional gene, while the red arrow on the top indicates the transcriptional direction of each functional gene.

Secondary metabolites play an important role in the cell viability of yeasts, including biocontrol yeasts such as *W. anomalus*, *Metschnikowia pulcherrima*, *Aureobasidium pullulans*, and *Saccharomyces cerevisiae* (Abdel-Kareem et al., 2019; Contarino et al., 2019). The prediction and annotation of protein-encoding genes in this study revealed that the genome of *C. oleophila* encodes a series of secondary metabolite genes. Among them, two distinct secondary metabolite clusters were identified using the antiSMASH online tool, a non-ribosomal peptide synthetase (NRPS)-like cluster and a terpenecluster. The NRPS-like and terpene clusters were composed of 18 and 9 functional genes, respectively (Figure 6). NRPS-like proteins are key enzymes in microorganisms that function in the assembly of peptide backbones of biologically-active natural products (Hühner et al., 2018). Terpenoids comprise a variety of compounds serving different functions in yeasts. For example, they facilitate attachment of proteins to membranes by thioether bonds in the form of prenyl-anchors (Wriessnegger and Pichler, 2013; Santiago-Tirado and Doering, 2016). The classification of various terpene synthases and their catalytic mechanisms have been recently reviewed (Gao et al., 2012). The antimicrobial activity of most terpenoids is linked to their functional groups, and it has been shown that the hydroxyl group of phenolic terpenoids and the presence of delocalized electrons are important for antimicrobial activity (Hyldgaard et al., 2012). For instance, a putative terpene cyclase, *vir4*, has been reported to be responsible for the biosynthesis of volatile terpene compounds in the biocontrol fugus, *Trichoderma virens*, thus contributing to its biocontrol efficacy (Crutcher et al., 2013). In the present study, we assume that the NRPS-like and terpene clusters within *C. oleophila* may play a role in their ability to attach to fungal and plant cell walls directly affecting its biocontrol efficacy. The ability of the biocontrol yeasts, *Pichia guilliermondii* and *Rhodotorula glutinis*, to attach to and parasitize the post-harvest pathogen *Botrytis cinerea* has also been reported (Wisniewski et al., 1991; Li et al., 2016).

## Conclusion

The genome of *C. oleophila* I-182, the active agent in the first-generation commercial yeast product Aspire^®^ developed for the biocontrol of post-harvest disease of fruits and vegetables was sequenced, assembled, and annotated. The genome size (14.73 Mb), along with the identification of CAZymes and secondary metabolite clusters, provides important genetic information on this biocontrol agent that can be used to better understand the various modes of action reported for this yeast, including competition for space and nutrients, hydrolysis of fungal cell walls, and induction of host disease resistance, at a molecular level. As the genome sequence of more biocontrol yeasts become available, it is hoped that the identification of “biocontrol” genes can be pursued. Such knowledge would help to identify traits that can be used to select effective biocontrol agents rather than by empirical selection methods alone.

## Data Availability Statement

The datasets generated for this study can be found in the PRJNA511409.

## Author Contributions

YS, XW, and JY conceived and designed the experiments and drafted the manuscript. YS, MW, SD, EP, and JY performed the experiments and analyzed the data. All authors read and approved the final manuscript.

## Conflict of Interest

The authors declare that the research was conducted in the absence of any commercial or financial relationships that could be construed as a potential conflict of interest.

## Footnotes

1 https://www.yeastgenome.org/

2 https://fungi.ensembl.org/

3 https://www.yeastgenome.org/

4 http://www.ncbi.nlm.nih.gov/bioproject/PRJNA511409

5 https://www.yeastgenome.org/
